# Bimetallic non-alloyed NPs for improving the broadband optical absorption of thin amorphous silicon substrates

**DOI:** 10.1186/1556-276X-9-181

**Published:** 2014-04-13

**Authors:** Chee Leong Tan, Sung Jun Jang, Young Min Song, Kamal Alameh, Yong Tak Lee

**Affiliations:** 1 Advance Photonic Research Institute, Gwangju Institute of Science and Technology, 1 Oryong-dong, Buk-gu, Gwangju 500-712, Republic of Korea; 2 School of Information and Communications, Gwangju Institute of Science and Technology, 1 Oryong-dong, Buk-gu, Gwangju 500-712, Republic of Korea; 3 Electron Science Research Institute, Edith Cowan University, 270 Joondalup Dr, Joondalup WA 6027, Australia; 4 Department of Electronics Engineering, Pusan National University, Busandaehak-ro, 63beon-gil, Geumjeong-gu, Busan 609-735, Republic of Korea

**Keywords:** Optics at surface, Surface plasmon resonance, Antireflection, Scattering light

## Abstract

We propose the use of bimetallic non-alloyed nanoparticles (BNNPs) to improve the broadband optical absorption of thin amorphous silicon substrates. Isolated bimetallic NPs with uniform size distribution on glass and silicon are obtained by depositing a 10-nm Au film and annealing it at 600°C; this is followed by an 8-nm Ag film annealed at 400°C. We experimentally demonstrate that the deposition of gold (Au)-silver (Ag) bimetallic non-alloyed NPs (BNNPs) on a thin amorphous silicon (a-Si) film increases the film's average absorption and forward scattering over a broad spectrum, thus significantly reducing its total reflection performance. Experimental results show that Au-Ag BNNPs fabricated on a glass substrate exhibit resonant peaks at 437 and 540 nm and a 14-fold increase in average forward scattering over the wavelength range of 300 to 1,100 nm in comparison with bare glass. When deposited on a 100-nm-thin a-Si film, Au-Ag BNNPs increase the average absorption and forward scattering by 19.6% and 95.9% compared to those values for Au NPs on thin a-Si and plain a-Si without MNPs, respectively, over the 300- to 1,100-nm range.

## Background

The deposition of metallic NPs (MNPs) on thin films has attracted great interest due to the ability of such NPs to enhance the optical absorption and scattering through the light-stimulated resonance of the conduction electrons within the NPs. Gold (Au) and silver (Ag) NPs have been widely used to (i) improve the absorption and reduce the total reflection of solar cells [1-4] and photodiodes [5], (ii) enhance the emission of light-emitting diodes (LEDs) [6,7], and (iii) increase the sensitivity of biosensors [8].

One of the key features of depositing MNPs onto the surface of optoelectronic devices is the ability of these NPs to control the localized surface plasmon resonance (LSPR) peak within a wavelength range of interest by simply varying the MNP type, size, shape, and spacing, and also by altering the dielectric medium surrounding the MNPs [2,9]. Various metal NP structures, such as single MNPs of various shapes (e.g., nanorod, triangular, sphere, star, etc.) [9], bimetallic core-shell NPs [10], and bimetallic alloy NPs [11], have been proposed for controlling the LSPR peak of optoelectronic devices. However, for such NP structures, light-stimulated resonance can only occur at specific wavelengths within a narrow wavelength range [1]. MNP-based structures having a narrow LSPR range are impractical for applications requiring broadband absorption, such as photovoltaic and optical telecommunications.

Motivated by the above-mentioned challenges, we propose in this paper the use of Au-Ag bimetallic non-alloyed NPs (BNNPs) to overcome the problem of narrowband absorption of single-type metal NPs; further, we experimentally demonstrate that such BNNPs exhibit LSPR peaks at 437 and 530 nm and enhance the average forward scattering ten times when deposited onto a glass substrate; when deposited on a 100-nm-thin a-Si film, the Au-Ag BNNPs increase the average absorption and forward scattering of the film by more than 85% over the wavelength range of 300 to 1,100 nm. We also verify that the lower total reflection is achieved only in Si films, because the bottom side of the Au-Ag BNNPs blocks the light reflected off the Si thin film/substrate interface and confines it within the Si film, whereas for a glass substrate, Au-Ag BNNPs significantly scatter the incident light, leading to higher total reflection.

## Methods

### Fabrication of BNNP nanostructures

**Figure 1:**
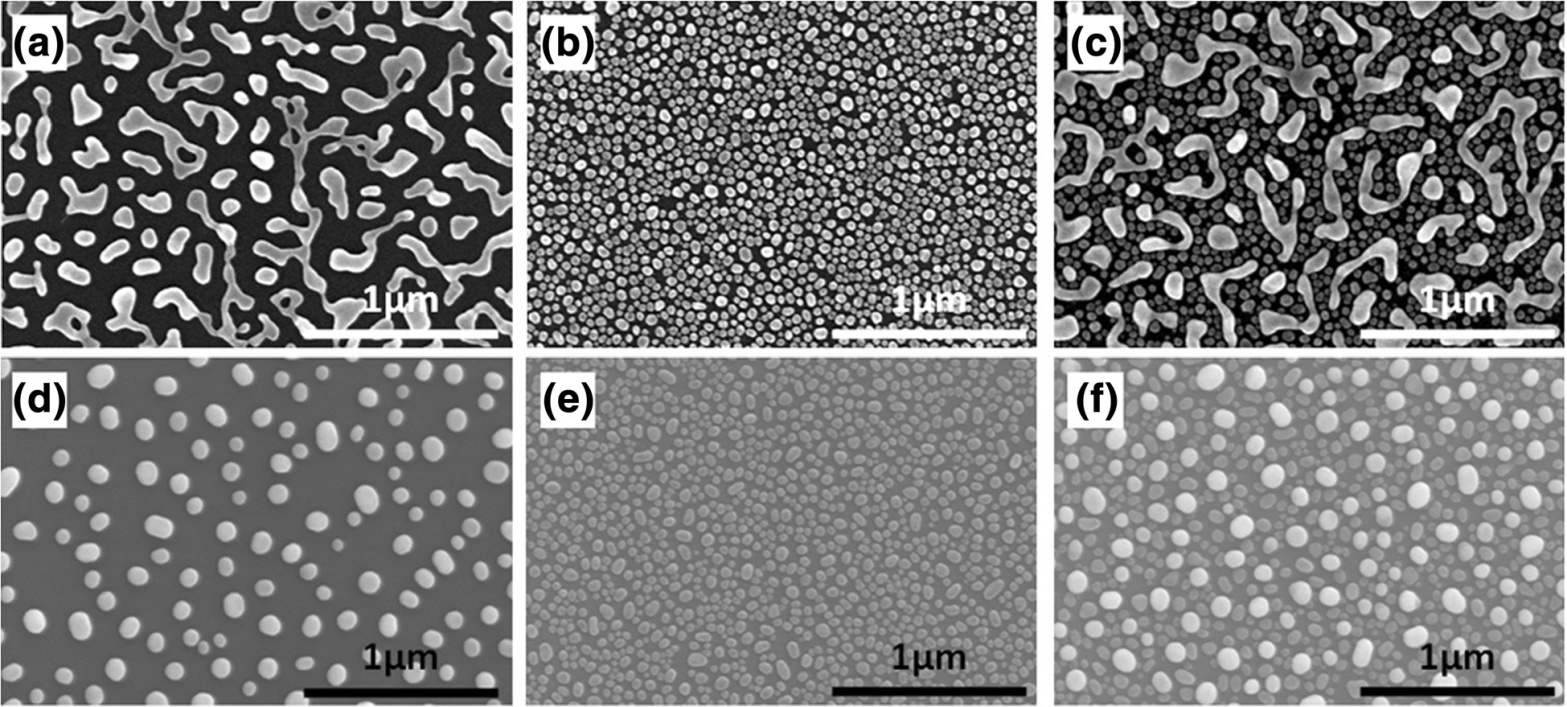
**SEM images of the BNNPs and NPs on thin a-Si film and glass substrates. (a, d)** Au MNPs on glass and thin a-Si film substrates, respectively (10-nm-thick Au film annealed at 600°C for 1 min). **(b, e)** Ag MNPs on glass and thin Si film substrates, respectively (8-nm-thick Ag film annealed at 400°C for 1 min). **(c, f)** Au-Ag BNNPs on glass and thin a-Si film substrates, respectively (10-nm-thick Au film annealed at 600°C for 1 min, followed by deposition of 8-nm-thick Ag film and annealing at 400°C for 1 min).

Au-Ag BNNPs were prepared using a modified two-step evaporation method that was originally used to prepare a compound metal island or alloyed MNPs [12]. In this study, three types of metal NP structures were synthesized on two different types of substrates, namely glass and thin a-Si films. Au-Ag BNNPs were deposited on a glass substrate to demonstrate their ability to increase the forward scattering of the BNNPs. First, glass substrates were cleaned sequentially using acetone, methanol, and iso-propanol solutions for 5 min each. All samples were also cleaned using a solution of diluted buffer oxide etchant (BOE) before the deposition of metal or thin a-Si. This was necessary in order to remove the native oxide on the surfaces of the samples. A 100-nm-thin a-Si film was initially deposited on one of the glass substrates using E-beam evaporation at a rate of 5 Ǻ s^-1^ under a pressure of 2 × 10^-6^ Torr. Au and Ag metals were chosen because they exhibit two different LSPR wavelengths (around 600 and 400 nm, respectively) that are significantly apart and because the difference in the melting temperatures for the two metals is substantial. A thin gold metal layer was deposited on a glass substrate with a low deposition rate in order to enhance the uniformity over a large surface. The thin Au metal was annealed at a temperature *T*_1_ = 600°C at which the Au NPs are clusterized. This clusterization can easily be noticed by comparing the scanning electron microscopy (SEM) images of the thin metal film before and after annealing. The thin metal film (originally flat) transforms into either hemisphere-shaped MNPs or a metal cluster, and both structures maintain the same shape even if the temperature is further increased up to a critical temperature, beyond which the metal particles melt and then evaporate. It should be noted that the impact of annealing on thin films has been well investigated by Müller et al. [13]. This step was used to prevent the gold thin film from mixing with the silver thin film, hence avoiding the formation of an alloy of MNPs. Then, a thin silver metal layer was deposited onto the Au NP system and annealed at temperature *T*_2_ (lower than *T*_1_), at which the Ag NPs crystallized. Figure 1 provides the SEM images of the three different metal NP systems. The Au NP systems shown in Figure 1a,d were synthesized on glass and thin a-Si films, respectively. These were achieved by initially depositing a thin Au metal film (10 nm) and annealing it at 600°C for 1 min. The difference in the shapes and sizes of the gold NPs on both glass and thin a-Si is due to the different levels of heat dissipation and the surface tension properties of the glass and thin a-Si films [13]. In Figure 1b,e, it can be seen that Ag NP systems were formed on glass and thin a-Si films, respectively, using an 8-nm-thick Ag film annealed at 400°C for 1 min. Finally, Au-Ag BNNPs, shown in Figure 1c,f, were synthesized on glass and thin a-Si films, respectively, using a 10-nm-thick Au film annealed at 600°C; this was followed by the deposition of an 8-nm-thick Ag thin film annealed at 400°C. These samples were characterized using a field emission SEM (S-4700, Hitachi, Chiyoda, Tokyo, Japan) operating at 10 kV, which enabled the study of the metal NP islands' size and distribution. Interestingly, Figure 1c,f demonstrates the ability of Au-Ag BNNPs to distribute evenly on glass and thin a-Si substrates. We can easily distinguish the Au NPs from the Ag NPs from their brightness and large size. Figure 1c,f demonstrates that the proposed fabrication process enables the formation of isolated non-alloyed NPs on glass and a-Si substrates and that both Au and Ag NPs can be crystallized. This is important because alloyed Au-Ag NPs only introduce a new LSPR peak but do not broaden the LSPR peak [12].

The average values for size, spacing, and surface density of the MNPs shown in Figure 1 are summarized in Table 1. These parameters were determined using the image processing software ImageJ [14], which can measure, over selected areas of the sample, NP sizes, mean, standard deviation, and min and max, and can then generate histograms and profile plots. The average spacing between the NPs was evaluated manually by determining the ‘nearest neighbor boundary’. It was noticed that the average NP size and spacing for single MNPs were not very different from those of bimetallic non-alloyed NPs. It was also found that when another batch of BNNPs was fabricated under similar conditions, the LSPR responses for the Au and Ag NPs were fairly similar for both batches, demonstrating the repeatability of the BNNP fabrication process.

**Table 1:** Summary of NP size distributions, spacing between particles, and surface densities

**Samples**	**NP diameter range in nm (mean)**		**NP to NP distance range in nm (mean)**			**Number of NPs on 245 × 169 nm (percentage of area coverage by NPs)**	
	**Au NPs**	**Ag NPs**	**Au-Au NPs**	**Ag-Ag NPs**	**Au-Ag NPs**	**Au NPs**	**Ag NPs**
Au NPs on glass	9.6 to 352.4 (130.5)		90.0 to 318.2 (193)			97 (37.9)	
Ag NPs on glass		7.8 to 111.7 (48.2)		45.4 to 118.2 (63.8)			1,451 (42.4)
AuAg NPs on glass	7.8 to 254.4 (124.5)	16.2 to 109.3 (43.8)	118.2 to 272.3 (180.9)	36.3 to 90.9 (58.48)	36.3 to 181.9 (61.2)	114 (23.5)	1,044 (25.6)
Au NPs on a-Si	13.5 to 162.4 (108.5)		90.0 to 363.4 (198)			135 (19.3)	
Ag NPs on a-Si		5.5 to 111.7 (52.2)		3.3 to 109 (62.2)			1,211 (42.4)
AuAg NPs on a-Si	37.6 to 105.1 (100.5)	7.8 to 126.8 (60.76)	127.3 to 290.1 (201.0)	45.5 to 118.2 (70.0)	36.3 to 145.5 (105)	149 (20.3)	544 (30.3)

## Results and discussion

**Figure 2:**
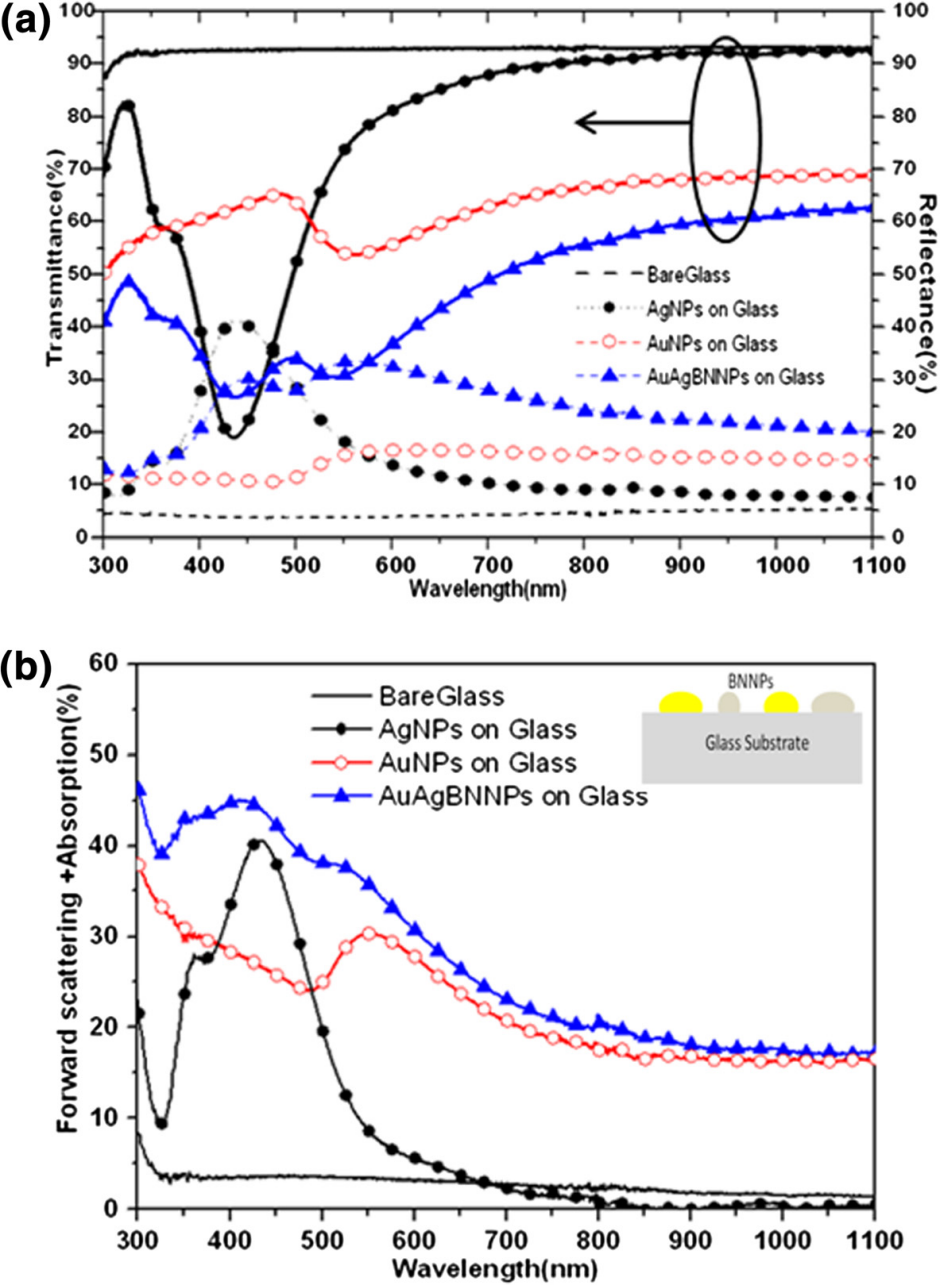
**Measured optical properties of Au NPs, Ag NPs, and Au-Ag BNNPs on glass substrate and bare glass (as a reference). (a)** Transmittance (solid line) and reflectance spectra (dot line) (the inset shows the BNNP structure on thin a-Si). **(b)** Forward scattering + absorption spectra.

Total reflection and transmission spectroscopy measurements were carried out to characterize the optical properties of the fabricated samples. Subsequently, the normalized optical absorption with forward scattering was calculated by subtracting the sum of normalized reflection and transmission from unity. The total reflectance and transmittance from all angles were measured over the wavelength range of 300 to 1,100 nm. The optical reflectance at all angles was obtained using a standard UV/vis-near-IR spectrophotometer (Cary 5000, Varian, Palo Alto, CA, USA) equipped with an integrated sphere. The transmittance was measured only for the normal incident angle, since measurement at normal incident angle was the only possible setup. Figure 2a,b shows the transmission, reflection, and calculated absorption with forward scattering spectra of the Au NPs, Ag NPs, and Au-Ag BNNPs deposited on glass substrates. The spectra for Au and Ag NPs are in excellent agreement with the spectra reported by Temple et al. [3] and Schaadt et al. [4]. Figure 2a shows that both the Au NPs and Ag NPs exhibit narrow LSPR peaks at 565 and 435 nm, respectively, whereas the Au-Ag BNNP sample displays LSPR peaks at 540 and 437 nm, which indicate higher average forward scattering, as shown in Figure 2b. Figure 2b clearly shows that forward scattering dominates when the glass substrate and the MNPs have minimum parasitic absorption. The forward scattering of Au-Ag BNNPs on glass is increased 1.2-fold, 3.0-fold, and 10.2-fold, respectively, compared to those values for Ag NPs on glass, Au NPs on glass, and bare glass structure.

**Figure 3:**
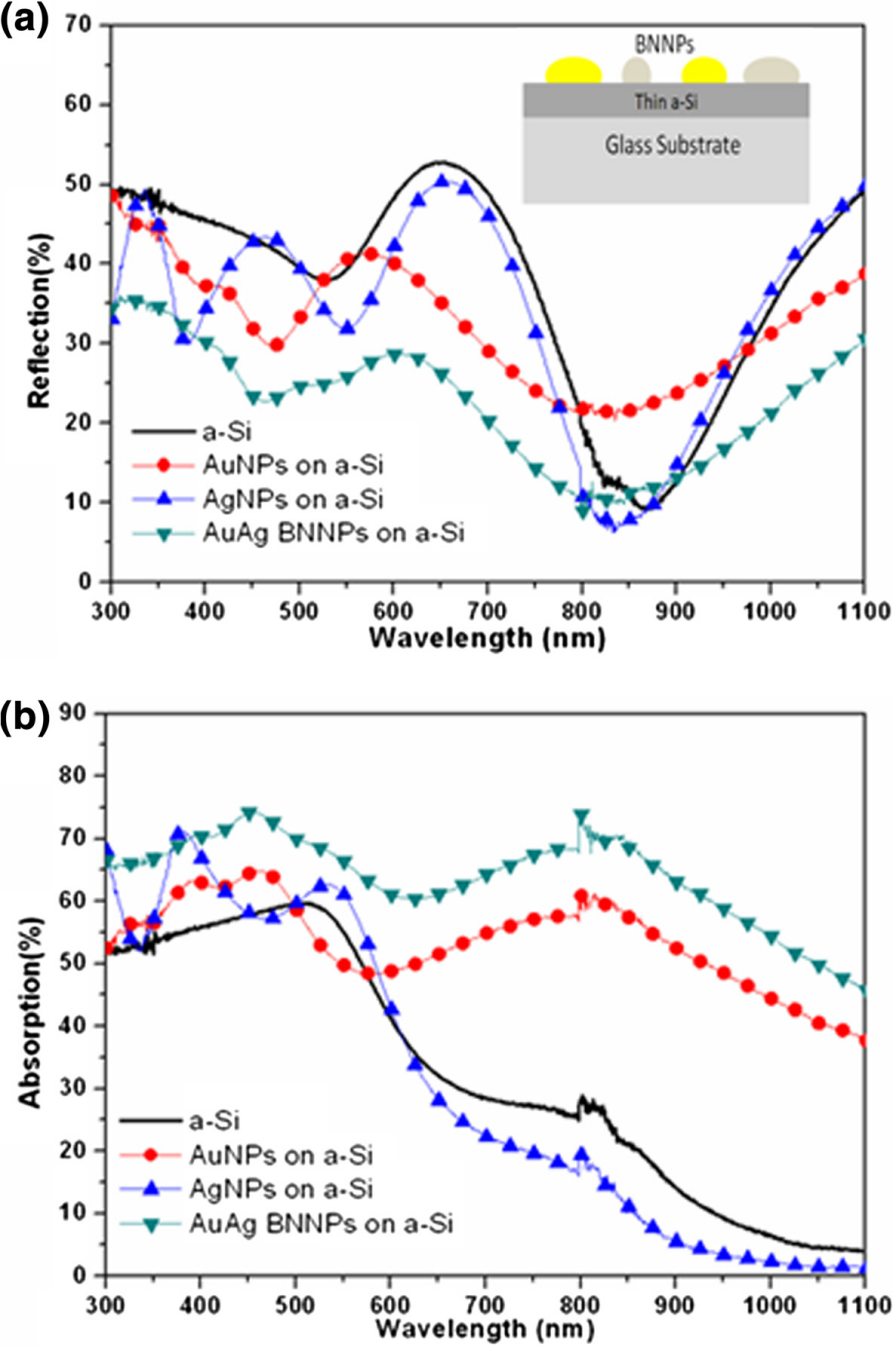
**Optical properties of Au NPs, Ag NPs, and Au-Ag BNNPs on thin a-Si films. (a)** Reflectance spectrum (the inset shows the BNNP structure on thin a-Si). **(b)** Absorption spectrum.

Figure 3a,b shows the measured reflection and calculated absorption spectra of Au NPs, Ag NPs, and Au-Ag BNNPs on thin a-Si films. The Ag and Au NP structures on thin a-Si film exhibit high absorption around 420 and 530 nm, respectively, and the wavelength span over which the absorption is enhanced is relatively narrow. However, it should be noticed that the absorption is slightly enhanced over the measured spectrum (300 to 1,100 nm) in comparison to the absorption of thin a-Si film. On the other hand, the average absorption and forward scattering of the Au-Ag BNNPs on thin a-Si films is at least 19.6% higher than that of Au NPs and at least 95.9% higher than that of plain a-Si without MNPs over the 300- to 1,100-nm range. As can be seen in Figure 3a, the deposition of MNPs lowers the reflection of amorphous Si, and thus these MNPs also act as antireflection structures. The average reflection of Au-Ag BNNPs is lower by 30.5%, 34%, and 39.5% compared to those values for Au NPs on a-Si, Ag NPs on a-Si, and Au-Ag BNNPs on a-Si, respectively. It should be noted that the Au and Ag NPs slightly reduce the reflection of thin a-Si films at around 420 and 530 nm, respectively. Au-Ag BNNPs, however, can achieve broadband antireflection due to the different average sizes of the Au and Ag NPs (average Au and Ag NP diameters are 100 and 60 nm, respectively). It should also be noted from Figures 2b and 3a that the reflection spectra of the MNPs deposited on the glass substrate differ from those fabricated on thin a-Si films. This discrepancy in reflection spectra can be explained through the diffusion model for light propagation [15]. When a light wave strikes a plain glass region, a fraction of it is reflected due to the air-glass interface; the remainder is transmitted. A glass substrate has a low refractive index, leading to low reflection from the top and bottom surfaces of the substrate. The direct reflection of the light off the MNPs on glass substrates is therefore the main phenomenon that leads to high reflection. On the other hand, the total reflection is reduced when the MNPs are deposited on thin a-Si films. This is because a large fraction of the light that is scattered into the thin Si film is reflected off its bottom surface due to the high refractive index of Si and also because the MNPs prevent light from escaping outside the thin film region, thus enhancing the film's absorption property over a broad wavelength band.

To investigate the effective absorption of the BNNPs on the solar cell performance under the solar radiation spectrum, we calculated the solar weighted absorption (SWA) enhancement, which can be explained as the ratio of absorption photons to total incident photons, i.e., the normalization of absorption spectra with the terrestrial air mass 1.5 global (AM 1.5G) [16], as given in the following equation [17]:

$$\mathrm{SWA}=\frac{\int A\left(\lambda \right)N_{\text{photon}}\mathrm{d\lambda}}{\int N_{\text{photon}}\mathrm{d\lambda}}$$

where *A*(*λ*) is the absorption and *N*_photon_ is the photon number of AM 1.5G per unit area per unit wavelength. The calculated solar weighted absorption (SWA) enhancement of AuNPs, Ag NPs, and Au-Ag BNNPs is summarized in Table 2. Table 2 clearly shows the disadvantages of single-type MNPs on a-Si layer. It was also noticed that Ag NPs have a lower SWA compared to that of a plain a-Si layer due to the higher reflection for mid-infrared wavelengths. This is explainable when we consider that the narrow LSPR resonance properties of Ag NPs only occurs for the visible wavelengths and that the backscattering of NPs at mid-infrared wavelengths increases the reflection of a-Si, as shown in Figure 3a. Table 2 shows that Au-Ag BNNPs on a-Si are a potential candidate for practical solar cells because they exhibit low broadband reflection and also high forward scattering, thus enhancing the SWA by 79% compared to that of plain thin a-Si for the wavelength range of 300 to 1,100 nm.

**Table 2:** Solar weighted absorption enhancement of Au NPs, Ag NPs, and Au-Ag BNNPs on thin a-Si substrates

**Samples**	**Average absorption**	**Solar weighted absorption (%)**	**SWA enhancement compared to plain a-Si (%)**
a-Si	32.56	36.33	
AuNPs on a-Si	53.40	54.27	49.3
AgNPs on a-Si	31.67	35.49	-2.0
AuAg BNNPs on a-Si	63.89	65.04	79.0

## Conclusions

We have presented a new approach to the fabrication of Au-Ag BNNPs, which can enhance the absorption of thin a-Si films through interparticle coupling and anti-reflection. A simple modified two-step evaporation process, enabling the deposition of Au-Ag bimetallic non-alloyed NPs using conventional micro-fabrication processes, has been described. Isolated Au-Ag bimetallic NPs with uniform size and spacing distribution have been deposited over large areas of glass and thin a-Si substrates. Experimental results have shown that the extinction bands of both the Au NPs and the Ag NPs deposited on glass substrates are narrow (200 to 400 nm) and that these materials exhibit resonance peaks at 565 and 435 nm, respectively. We also found that the Au-Ag BNNPs display two LSPR peaks at 437 and 540 nm; they have higher overall absorption coefficients. It was also shown that the average absorption and forward scattering of the Au-Ag BNNPs on thin a-Si increased by 19.6% and 95.9% compared to those values for Au NPs on thin a-Si and plain a-Si without MNPs, respectively, over the 300- to 1,100-nm range. These results will find application in Si photovoltaics and optical telecommunications.

## Competing interests

The authors declare that they have no competing interests.

## Authors’ contributions

CLT carried out the experimental work associated with the fabrication and characterization of the samples, analyzed the results, and prepared the manuscript. YMS and SJJ helped in the analysis of the results and preparation of the manuscript. KA helped prepare the manuscript. YTL developed the conceptual framework and supervised the whole project, including finalizing the manuscript. All authors read and approved the final manuscript.
